# IFN-*γ* Mediates the Development of Systemic Lupus Erythematosus

**DOI:** 10.1155/2020/7176515

**Published:** 2020-10-17

**Authors:** Wenping Liu, Mengdi Li, Ziye Wang, Jibo Wang

**Affiliations:** Department of Rheumatoid and Immunology, The Affiliated Hospital of Qingdao University, Qingdao 266000, China

## Abstract

**Objective:**

Systemic lupus erythematosus (SLE) is a chronic autoimmune disease that can affect all organs in the body. It is characterized by overexpression of antibodies against autoantigen. Although previous bioinformatics analyses have identified several genetic factors underlying SLE, they did not discriminate between naive and individuals exposed to anti-SLE drugs. Here, we evaluated specific genes and pathways in active and recently diagnosed SLE population.

**Methods:**

GSE46907 matrix downloaded from Gene Expression Omnibus (GEO) was analyzed using R, Metascape, STRING, and Cytoscape to identify differentially expressed genes (DEGs), enrichment pathways, protein-protein interaction (PPI), and hub genes between naive SLE individuals and healthy controls.

**Results:**

A total of 134 DEGs were identified, in which 29 were downregulated, whereas 105 were upregulated in active and newly diagnosed SLE cases. GO term analysis revealed that transcriptional induction of the DEGs was particularly enhanced in response to secretion of interferon-*γ* and interferon-*α* and regulation of cytokine production innate immune responses among others. KEGG pathway analysis showed that the expression of DEGs was particularly enhanced in interferon signaling, IFN antiviral responses by activated genes, class I major histocompatibility complex (MHC-I) mediated antigen processing and presentation, and amyloid fiber formation. STAT1, IRF7, MX1, OASL, ISG15, IFIT3, IFIH1, IFIT1, OAS2, and GBP1 were the top 10 DEGs.

**Conclusions:**

Our findings suggest that interferon-related gene expression and pathways are common features for SLE pathogenesis, and IFN-*γ* and IFN-*γ*-inducible GBP1 gene in naive SLE were emphasized. Together, the identified genes and cellular pathways have expanded our understanding on the mechanism underlying development of SLE. They have also opened a new frontier on potential biomarkers for diagnosis, biotherapy, and prognosis for SLE.

## 1. Introduction

Systemic lupus erythematosus (SLE) is a chronic autoimmune disease that can affect all organs in the body. It is characterized by hyperactivation of the immune system, resulting in abnormal production of autoantibodies, particularly antinuclear antibodies [1]. The clinical manifestations and severity of the disease are diverse, but are usually life-threatening. Currently, the global incidence of SLE is about 40/100,000 [2]. Women in their reproductive years are the most susceptible group for the development of SLE. The pathogenesis of SLE is complex, and the whole pathogenesis cascade is mediated by immune disorders. A lot of effort has been made to study the pathogenesis of SLE. In effect, hereditary, environmental, and hormonal dynamics have been identified as three major factors in SLE etiopathogenesis [3]. However, genetic factors are the major variables that mediate the pathogenesis of SLE [4]. Nonetheless, specific genes and pathways mediating the pathogenesis of the disease are not well understood. This in effect has undermined progress in treatment and prognosis prediction of SLE.

High-throughput sequencing is a new omics technology that has opened up a new frontier in disease genomics [5]. In the recent past, the combination of microarray technology and bioinformatics has facilitated the understanding of molecular mechanisms and reliable diagnostic and therapeutic targets for diseases. Through these techniques, many new genes involved in complex human diseases such as cancer and autoimmune complications have also been discovered [6, 7]. Several studies have identified disease-related genes and functional pathways by analyzing the expression profile between SLE and healthy controls [8–12]. However, in the majority of the studies, SLE patients have a history of exposure to anti-SLE drugs, a variable that can cause considerable transcriptional changes [13]. Consequently, findings from such studies may mask some features that would have otherwise been apparent. Therefore, in order to eliminate the interference of drugs on transcriptomes, the GSE46907 dataset from NCBI-GEO encompassed 5 newly diagnosed SLE patients without history of exposure to anti-SLE drugs and 5 controls was selected. Peripheral monocyte transcript samples were then obtained from the identified individuals for evaluation. Differentially expressed genes between the two groups were identified and analyzed based on the principles of Gene Ontology (GO) and Kyoto Encyclopedia of Genes and Genomes (KEGG) pathway. Protein-protein interaction (PPI) network of the DEGs and hub genes was constructed using Cytoscape. Our study is aimed at identifying dominant genes associated with the pathogenesis of naive SLE in view of providing new targets and knowledge for the diagnosis and treatment of the disease.

## 2. Materials and Methods

### 2.1. Affymetrix Microarray Data

GSE46907 gene expression profile for peripheral blood monocytes both from SLE patients and controls was downloaded from the Gene Expression Omnibus database (available at https://www.ncbi.nlm.nih.gov/geo/) [14]. This dataset included gene expression profiles for 5 pediatric SLE patients naive for anti-SLE drugs. The matrix was based on GPL96 platforms (HG_U133A; Affymetrix Human Genome U133A Array).

### 2.2. Identification of Differentially Expressed Genes (DEGs)

DEGs between SLE patients and controls were identified using the LIMMA package downloaded from Bioconductor (http://www.bioconductor.org/packages/release/bioc/html/limma.html) in R software (version 3.4.0; https://www.r-project.org/). Student's *t*-test in the LIMMA package was used to analyze significant difference between the groups. DEGs with adjusted *P* < 0.05 and ∣log2 fold change | >1 were considered to be statistically significant. The genetic profiles for the peripheral blood monocytes were visualized using a heat map of hierarchical cluster analysis (HCA) and volcano plot.

### 2.3. Gene Ontology and Pathway Enrichment Analyses of DEGs

GO (Gene Ontology) terms [15] and KEGG (Kyoto Encyclopedia of Genes and Genomes) pathway enrichment analyses are two widely used approaches in understanding high-level utilities of biological systems in bioinformatics research [16]. Metascape (http://metascape.org) was used to perform functional enrichment analysis. This is an online analytical tool integrated with several ontology sources including the KEGG pathway, GO biological processes, Reactome gene sets, canonical pathways, and CORUM [17]. Pathway and process enrichment for each DEGs gene were analyzed for statistical significance in each biological process. Only terms with *P* < 0.01, a minimum count of 3 and an enrichment factor > 1.5 were considered significant. Genes were clustered according to their pathways. Bar charts were used to visualize the results for the GO terms and KEGG pathway enrichment analyses of the DEGs.

### 2.4. PPI Network Analysis and Hub Gene

The STRING online database (http://string-db.org) predicted the protein-protein interaction (PPI) [18] between DEGs. A combined score > 0.4 was considered significant. The PPI network was visualized using Cytoscape software (version 3.4.0; http://www.Cytoscape.org). It simplified the complex network of PPI. Nodes with a high degree from the Network Analyzer plugin (cytoHubba) [19] were classified as hub genes. These are genes potentially mediating key physiological regulatory functions in SLE.

## 3. Results

### 3.1. DEGs

**Figure 1:**
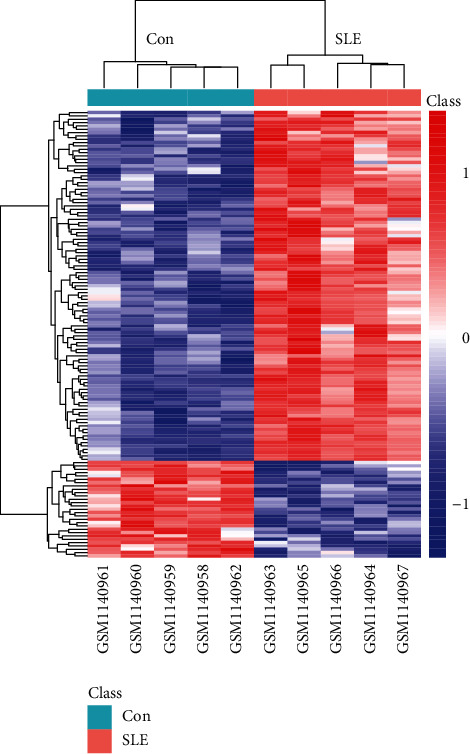
Heat map of hierarchical clustering analysis for the DEGs between SLE and healthy controls. Each row represents a gene, and each column represents a sample. Color indicates the level of gene expression. Red represents high expression, and green represents low expression. The top is the sample cluster tree, and the left is the gene cluster tree.

**Figure 2:**
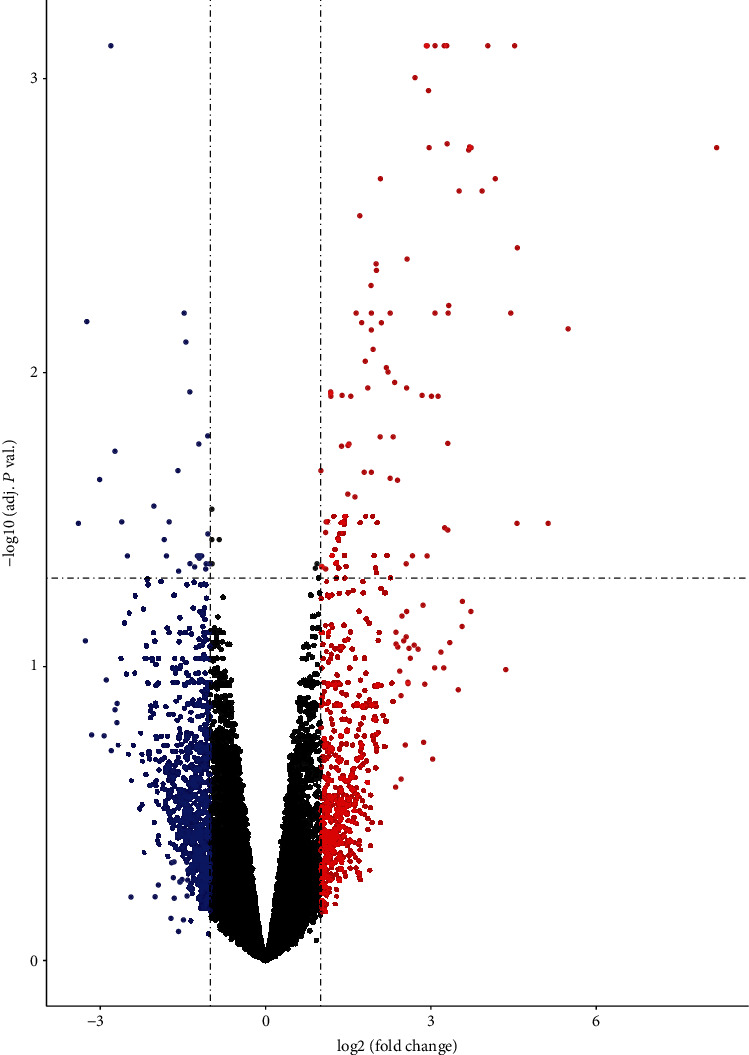
The volcano plots of genes in SLE and healthy control. The vertical lines represent |log2.0 fold change| of SLE/healthy control up and down, respectively, and the horizontal line represents adj. *P* value of 0.05. And in the plot, red and blue points represent magnitude change of DGEs for SLE and healthy control, respectively.

This study is aimed at identifying DEGs between normal and individuals with SLE. Given that exposure to anti-SLE drugs can interfere normal transcription, the selected SLE patients in GSE46907 were healthy, newly diagnosed with the disease, and naive for autoimmune medications. The expressed transcripts in SLE peripheral blood monocytes were obtained and analyzed. Compared with healthy control, a total of 134 DEGs were identified in individuals with SLE, in which 29 were downregulated, whereas 105 were upregulated. The heat map and volcano plot for the DEGs are shown in Figures 1 and 2, respectively. Further details on the DEGs between the two groups are shown in Supplementary Materials in Table 1.

### 3.2. Gene Ontology and Pathway Enrichment Analyses of DEGs

**Figure 3:**
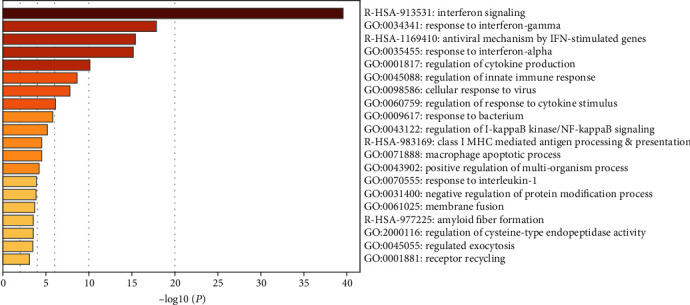
Top 20 enriched terms of function enrichment analysis of the DEGs identified in SLE, analyzed by Metascape.

Signaling pathway enrichment analysis of DEGs was performed using Metascape. The top 20 clusters for enriched sets are shown in Figure 3. For GO term enrichment analysis, expression of DEGs was mainly enhanced in response to secretion of interferon-*γ* and interferon-*α*, regulation of cytokine production, regulation of innate immune response, cellular viral responses, and regulation of I-kappaB kinase/NF-kappaB signaling. KEGG analysis revealed that the expression of DEGs was mostly enhanced in interferon signaling, antiviral responses by IFN-stimulated genes, and class I major histocompatibility complex (MHC-I) mediated antigen processing and presentation and formation of amyloid fibers. Specific genes associated with each pathway are shown in Table 1.

### 3.3. PPI Network Analysis and Hub Genes

**Figure 4:**
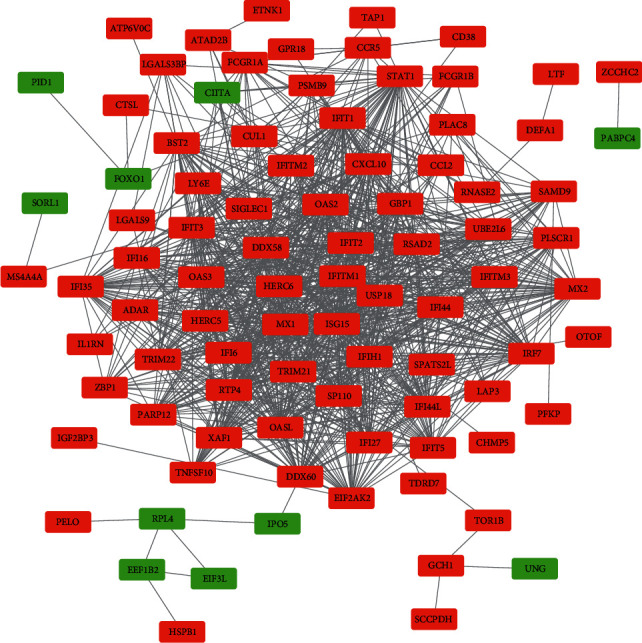
Protein-protein interaction (PPI) network of DEGs constructed using the STRING online database and Cytoscape 3.7.2, with 90 nodes and 973 edges. Red represents upregulated genes, while green represents downregulated genes.

**Figure 5:**
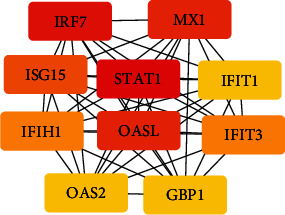
Top 10 hub genes and cointeraction in the PPI network, constructed by cytoHubba of Cytoscape based on a degree score. Color scale represents highly of degree scores.

A PPI network for the DEGs was constructed on the STRING website. The results were then visualized using Cytoscape (Figure 4). Generally, there were 80 nodes and 947 edges in the PPI network. Most DEGs in the PPI network were upregulated, with only six found to be downregulated. The top 10 genes with the highest degrees in the PPI network were identified using a plugin tool (cytoHubba) in the Cytoscape. They were as follows: signal transducer and activator of transcription 1 (STAT1, degree: 54), interferon regulatory factor 7 (IRF7, degree: 50), MX dynamin-like GTPase 1 (MX1, degree: 49), 2′-5′-oligoadenylate synthetase-like (OASL, degree: 49), ISG15 ubiquitin-like modifier (ISG15, degree: 48), interferon-induced protein with tetratricopeptide repeats 3 (IFIT3, degree: 47), interferon induced with helicase C domain 1 (IFIH1, degree: 47), interferon-induced protein with tetratricopeptide repeats 1 (IFIT1, degree: 46), 2′-5′-oligoadenylate synthetase 2 (OAS2, degree: 46), and guanylate binding protein 1 (GBP1, degree: 46). The interactions of the top 10 hub genes are shown in Figure 5. Detailed information on the listed genes and their functions is shown in Table 2.

## 4. Discussion

SLE is a heterogeneous autoimmunity disease characterized by overexpression of antibodies against autoantigens [20]. Genetic factors play critical roles in the development of SLE [21]. The role of single and cluster genes in the development and pathogenesis of SLE is described [22]. Recent technological advancements such as GWAS and microarray have expanded our understanding on gene expression, including those associated with the pathogenesis of SLE [23]. The previous studies however focused on the expression signature of blood IFN-*α* [24–26]. Even so, the majority of individuals sampled for most of these studies did not discriminate naive and anti-SLE-exposed individuals, a factor that may influence gene transcription profile. This study regularized this limitation. GSE46907 matrix was downloaded from the GEO databases analyzed with integrated bioinformatics methods with a view of identifying reliable genes and pathway associated with SLE. Notably, all patients involved were naive for anti-SLE drug therapy. In the end, 134 DEGs were identified; most of which were upregulated. The upregulated category consisted of genes involved in the activation of the immune system rather than immunosuppression. Among them, 90 DEGs (10 downregulated, 80 upregulated) constituted a PPI network. Further analysis using Cytoscape revealed top 10 hub genes: STAT1, IRF7, MX1, OASL, ISG15, IFIT3, IFIH1, IFIT1, OAS2, and GBP1. Hub genes may mediate the development of SLE; thus, are potential targets and biomarkers for disease treatment and prognosis, respectively.

Previous bioinformatics analysis showed that IFN signature and IFN signaling pathways against viral infections were strongly associated with the pathogenesis and development of SLE. High levels of serum IFN together with overexpression of IFN-inducible genes have been found in individuals with SLE. The level of IFN correlated with the severity of the disease [12]. The role of type I IFN, paticularly IFN-*α* in the development and pathogenasis of SLE has been described [26]. To a greater extent, findings of this study were consistent with reports from previous studies. First, our analysis revealed that innate IFNs against viral infection were critical in the development and or pathogenesis of SLE. The overall interferon pathway was the most enhanced pathway in the pathogenesis of SLE. The top 10 hub genes identified in our studies are all associated with excessive production of IFN in viral infection and innate immune response (detailed in Table 2). Interestingly, 9 (STAT1, IRF7, MX1, OASL, ISG15, IFIT3, IFIH1, IFIT1, and OAS2) out of the 10 hub genes have been reported to be associated with SLE (except GBP1) previously [4, 11, 27, 28]. Second, class I MHC-mediated antigen processing and presentation and I-kappaB kinase/NF-kappaB signaling pathway were also critical in our study. One study found that class I MHC was strongly expressed among SLE individuals [29]. In 16/6ld-immunized mouse models, MHC molecules mediated induction of SLE, whereas repressing transcription of class I MHC prevents clinical manifestations of SLE [30]. Interestingly, the expression of MHC I molecules is enhanced by type I IFN [31]. Here, the interplay between type I IFN and MHC I molecules enhances peptide recognition by cytotoxic T cells. This intern promotes the induction of cell-mediated SLE. Similarly, the NF-*κ*B signaling pathway also participates in regulating the immune and inflammatory responses in individuals with SLE [32]. Therefore, inhibiting the NF-*κ*B signaling pathway could ameliorate symptoms of lupus nephritis or lupus-prone MRL/lpr mice [33, 34]. Because our study supports previous findings, it further strengthens our understanding on the pathogenesis of SLE.

Remarkably, our findings underscored the importance of IFN-*γ* in the pathogenesis of innate SLE, as opposed to IFN-*α* which has been emphasized in the previous bioinformatics analysis. IFN-*γ* is the only member of type II interferons, mainly produced by Th1-type T cells and NK cells [35]. One study found that in SLE patients, increase in the secretion of IFN-*γ* and IFN-*α* was positively correlated, and that types I and II IFN partially share signal pathways and target genes [36]. This crossinteraction facilitates coordination of these IFNs in executing specific functions in the immunopathogenesis of the SLE. Besides this, Groettrup et al. found that IFN-*γ* crossinteracts with MHC molecules, enhancing the quantity, quality, and repertoire of peptides bound by both class I and class II MHC [37]. IFN-*γ* can activate the transcription of both class I and class II MHC molecules, which later contribute to the development and severity of SLE. In one study, it was found that secretion of IFN-*γ* was elevated before the production of IFN-*α* and lupus-associated autoantibodies [35]. Because IFN-*γ* links innate and adaptive immunity, it can drive secretion of both type I IFN and BLyS (B lymphocyte stimulator) [38]. Chodisetti et al. found that IFN-*γ* is indispensable for TLR7-promoted development of autoreactive B cells and systemic autoimmunity [39]. IFN-*γ* can also regulate the production of Ig immunoglobulins and B cell class switching [40]. One study found that IFN-*γ* can promote aberrant activation and imbalanced polarization of macrophages [41], which also play an important role in SLE [42]. From a bioinformatics perspective, our study solidified the role of IFN-*γ* in the development of SLE. Based on previous bioinformatics analyses, we hypothesized that use of drugs such glucocorticoids may mainly reduce the initial IFN-*γ* signature. According to our analyses, one new hub gene (GBP1) that participates in the innate SLE was discovered. GBP1 is a critical interferon-stimulated gene (ISG). Interferon stimulation, particularly IFN-*γ*, activates overexpression of this gene [43]. Some studies suggest that GBP1 can be used as a marker for IFN-*γ* response [44]. GBP1 gene product can inhibit cellular proliferation and apoptosis in the early cellular inflammatory response [45]. GBP1 is also essential in intracellular defense in varied settings such as in oxidative or other cellular damages [46]. Furthermore, special attention has been put on the role of GBP1 in cancer pathology [47]. GBP1 is a potential target for cancer inflammatory responses [46]. In our case, there are limited researches on the relationship between GBP1 and SLE. However, our study underscores the significant role played by GBP1 and IFN-*γ* in the early onset of SLE; thus, the role of GBP1 on SLE should be further investigated.

## 5. Conclusion

In conclusion, this study strengthens the critical role played by IFN signatures and IFN signaling pathway in SLE. Different from previous bioinformatics analyses, we analyzed the transcription profile of treatment of the naive SLE patients. We found that IFN-*γ* and its response GBP1 genes play critical roles at the onset of SLE. These findings gave a new insight in the pathogenesis of the disease. It provided valuable markers or therapeutic targets for GBPI. However, this study had several limitations. First, the sample size used in our analysis was small, reducing the reliability of our findings. Second, given that some studies found that the expression of IFN-*γ* in peripheral blood mononuclear cells (PBMCs) in individuals with SLE was high and the dataset selected for this research measured the transcription profiles of peripheral blood monocyte, our findings may be biased. Therefore, our results require further validation.

## Figures and Tables

**Table 1:** Pathway enrichment analysis of SLE-associated DEGs using Metascape.

Pathway enrichment analysis	Genes
R-HSA-913531: interferon signaling	HSPB1, RSAD2, OAS2, OASL, IFIT1, HERC5, OAS3, IFIT3, ISG15, IFI27, IFI44L, IFITM1, MX2, USP18, IFI44, CUL1, RTP4, IFI6, IFI35, MX1, EIF2AK2, IFIH1, LTF, XAF1, IFITM2, DDX60, IFIT5, IFIT2, TRIM22, CHMP5, PLSCR1, IFITM3, PSMB9, LHFPL2, UBE2L6, TRIM34, IRF7, ZBP1, BST2, IL1RN, RNASE2, STAT1, FCGR1B, IFI16, DEFA1, TRIM21, DDX58, CCL2, CCR5, STAP1, OPTN, CXCL10, ADAR, PLAC8, FOXO1, LGALS9, FCGR1A, CIITA, GBP1
GO:0034341: response to interferon-gamma	RSAD2, OAS2, OASL, IFIT1, OAS3, IFITM1, CUL1, MX1, EIF2AK2, IFIH1, TAP1, IFITM2, TRIM22, IFITM3, TRIM34, IRF7, BST2, STAT1, FCGR1B, TNFSF10, IFI16, TRIM21, DDX58, CCL2, GCH1, CXCL10, ADAR, LGALS9, FCGR1A, CIITA, GBP1
R-HSA-1169410: Antiviral mechanism by IFN-stimulated genes	RSAD2, OAS2, OASL, IFIT1, HERC5, AS3, ISG15, MX2, USP18, MX1, EIF2AK2, IFIH1, LTF, DDX60, GPR18, UBE2L6, IRF7, BST2, STAT1, DDX58, ANXA4, OPTN, LGALS9, GBP1
GO:0035455: response to interferon-alpha	IFIT1, IFIT3, IFITM1, MX2, EIF2, AK2, XAF1, IFITM2, IFIT2, PLSCR1, IFITM3, BST2, STAT1, IFI16, DEFA1, TRIM21, CCR5, ADAR, LGALS9
GO:0001817: regulation of cytokine product	HSPB1, RSAD2, HERC5, ISG15, EIF2AK2, IFIH1, LTF, DDX60, SORL1, GPR18, UBE2L6, IRF7, ZBP1, BST2, STAT1, IFI16, TRIM21, DDX58, IGF2BP3, ANXA4, LGALS9, GBP1
GO:0045088: regulation of innate immune response	RSAD2, USP18, CUL1, IFIH1, LTF, DDX60, LSCR1, PSMB9, IRF7, ZBP1, STAT1, FCGR1B, IFI16, DDX58, CTSL, STAP1, OPTN, ADAR, LGALS9, FCGR1A, GBP1, CD38
GO:0098586: cellular response to virus	HSPB1, RSAD2, NR2F6, IFIT1, ISG15, IFI27, IFI6, IFIH1, LTF, DDX60, SORL1, GPR18, IPO5, IRF7, ZBP1, IL1RN, STAT1, DEFA1, DDX58, CCL2, CCR5, ANXA4, CXCL10, ADAR, FOXO1, LGALS9, NR1D2, GBP1, CD38
GO:0060759: regulation of response to cytokine stimulus	SP18, IFIH1, IRF7, ZBP1, IL1RN, STAT1, DDX58, STAP1, CXCL10, ADAR
GO:0009617: response to bacterium	HSPB1, NR2F6, OAS2, ISG15, IFITM1, IFI44, LTF, DDX60, CHMP5, HERC6, GPR18, RHOB, BST2, IL1RN, RNASE2, IFI16, PID1, DEFA1, TRIM21, CCL2, CCR5, STAP1, GCH1, OPTN, CXCL10, ADAR, PLAC8, FOXO1, LGALS9, NR1D2
GO:0043122: regulation of I-kappaB kinase/NF-kappaB signaling	HSPB1, EIF2AK2, LTF, ARID5B, IFIT5, TRIM22, GPR18, TRIM34, BST2, STAT1, TNFSF10, RIM21, DDX58, ANXA4, OPTN, LGALS9
R-HSA-983169: class I MHC-mediated antigen processing and presentation	HERC5, IFI27, SIGLEC1, IFITM1, CUL1, RASGRP3, TAP1, PLSCR1, PSMB9, HERC6, UBE2L6, FCGR1B, TRIM21, CTSL, STAP1, ATP6V0C, FCGR1A, GBP1, CD38
GO:0071888: macrophage apoptotic process	IRF7, CTSL, CCR5, LGALS9
GO:0043902: positive regulation of multiorganism process	IFIT1, DDX60, LHFPL2, TRIM21, OPTN, ADAR, LGALS9
GO:0070555: response to interleukin-1	UL1, RASGRP3, PSMB9, IL1RN, STAT1, PID1, CCL2, CCR5, GCH1, CXCL10, FOXO1, H2AC18, H2AC19, LGALS9, GBP1, CD38
GO:0031400: negative regulation of protein modification process	HSPB1, IFIT1, ISG15, IFIH1, SORL1, CHMP5, IPO5, SKI, PRR7, STAT1, TNFSF10, PID1, TRIM21, DDX58, STAP1, ATP6V0C, ADAR, FOXO1, CRTAP, GBP1
GO:0061025: membrane fusion	MX2, MX1, TAP1, SAMD9, CCR5, OTOF
R-HSA-977225: amyloid fiber formation	USP18, IFIH1, LTF, SORL1, PSMB9, UBE2L6, TRIM21, DDX58, UNG, H2AC18, H2AC19, FCGR1A
GO:2000116: regulation of cysteine-type endopeptidase activity	HSPB1, ISG15, IFI27, CUL1, IFI6, LTF, SORL1, PSMB9, IRF7, PRR7, BST2, STAT1, TNFSF10, IFI16, ADAR, PABPC4, LGALS9, CD38
GO:0045055: regulated exocytosis	LGALS3BP, IFI27, EIF2AK2, ADGRE3, LTF, PLSCR1, LHFPL2, BST2, RNASE2, DEFA1, DDX58, ENPP4, OTOF, STAP1, UNG, ATP6V0C, OPTN, CXCL10, PLAC8, LGALS9, SCCPDH
GO:0001881: receptor recycling	MX2, MX1, SORL1, CHMP5, OPTN

**Table 2:** Top 10 hub genes with high degree of DEGs in SLE.

Gene symbol	Full name	Function	Degree
STAT1	Signal transducer and activator of transcription	Signal transducer and transcription activator that mediates cellular responses to interferons (IFNs), cytokine KITLG/SCF, and other cytokines and other growth factors. Gene Ontology (GO) annotations related to this gene include DNA-binding transcription factor activity and protein homodimerization activity.	54
IRF7	Interferon regulatory factor	Key transcriptional regulator of type I interferon- (IFN-) dependent immune responses and plays a critical role in the innate immune response against DNA and RNA viruses. Gene Ontology (GO) annotations related to this gene include DNA-binding transcription factor activity.	50
MX1	MX dynamin-like GTPase 1	Interferon-induced dynamin-like GTPase with antiviral activity against a wide range of RNA viruses and some DNA viruses. Gene Ontology (GO) annotations related to this gene include GTP binding and GTPase activity.	49
OASL	2′-5′-Oligoadenylate synthetase-like	Does not have 2′-5′-OAS activity, but can bind double-stranded RNA. Displays antiviral activity against encephalomyocarditis virus (EMCV) and hepatitis C virus (HCV) via an alternative antiviral pathway independent of RNase L. Gene Ontology (GO) annotations related to this gene include double-stranded RNA binding.	49
ISG15	ISG15 ubiquitin-like modifier	Ubiquitin-like protein which plays a key role in the innate immune response to viral infection either via its conjugation to a target protein (ISGylation) or via its action as a free or unconjugated protein. Gene Ontology (GO) annotations related to this gene include protein tag.	48
IFIT3	Interferon-induced protein with tetratricopeptide repeats 3	IFN-induced antiviral protein which acts as an inhibitor of cellular as well as viral processes, cell migration, proliferation, signaling, and viral replication. Gene Ontology (GO) annotations related to this gene include identical protein binding.	47
IFIH1	Interferon induced with helicase C domain 1	Innate immune receptor which acts as a cytoplasmic sensor of viral nucleic acids and plays a major role in sensing viral infection and in the activation of a cascade of antiviral responses including the induction of type I interferons and proinflammatory cytokines. Gene Ontology (GO) annotations related to this gene include nucleic acid binding and hydrolase activity.	47
IFIT1	Interferon-induced protein with tetratricopeptide repeats 1	Interferon-induced antiviral RNA-binding protein that specifically binds single-stranded RNA bearing a 5′-triphosphate group (PPP-RNA), thereby acting as a sensor of viral single-stranded RNAs and inhibiting expression of viral messenger RNAs. Gene Ontology (GO) annotations related to this gene include RNA binding.	46
OAS2	2′-5′-Oligoadenylate synthetase 2	Interferon-induced, dsRNA-activated antiviral enzyme which plays a critical role in cellular innate antiviral response. Gene Ontology (GO) annotations related to this gene include RNA binding and transferase activity.	46
GBP1	Guanylate binding protein 1	Hydrolyzes GTP to GMP in 2 consecutive cleavage reactions. Exhibits antiviral activity against influenza virus. Promotes oxidative killing and delivers antimicrobial peptides to autophagolysosomes, providing broad host protection against different pathogen classes. Gene Ontology (GO) annotations related to this gene include identical protein binding and enzyme binding.	46

## Data Availability

The data used for analysis in this study are available from the Gene Expression Omnibus database freely.

## Supplementary Materials

Supplementary Materials Supplementary Materials of Table 1: the results of differentially expressed genes (DEGs) of healthy and newly diagnosed SLE population.
